# An Uncommon and Severe Clinical Case of *Sarcoptes scabiei* Infestation in a Cat

**DOI:** 10.3390/pathogens12010062

**Published:** 2022-12-30

**Authors:** Mariasole Colombo, Simone Morelli, Marzia Sacra, Gaia Trezza, Barbara Paoletti, Donato Traversa, Angela Di Cesare

**Affiliations:** 1 Department of Veterinary Medicine, University of Teramo, 64100 Teramo, Italy; 2 Clinica Veterinaria Borghesiana, 00132 Rome, Italy

**Keywords:** sarcoptic mange, mites, skin lesions, zoonosis

## Abstract

The burrowing mite *Sarcoptes scabiei* causes sarcoptic mange in dogs and other mammals, including humans. Despite *S. scabiei* infests several animals, little is known about the epidemiology, clinical features, and treatment of sarcoptic mange in cats. A few reports have shown that clinical signs of *S. scabiei* infestation in cats may vary from non-pruritic crusted lesions to itchy and mild lesions, while severe signs are very infrequent. The present report describes a severe case of *S. scabiei* infestation in a stray cat from Italy, characterized by bilateral alopecia, diffuse and multifocal hyperpigmented, crusted, erythematous, scaled, and exfoliated lesions. The cat was FIV-positive and also infected by the cestode *Dipylidium caninum*. After treatment with a broad-spectrum parasiticide and an antibiotic, the cat showed an almost complete clinical recovery in 4 weeks. Unfortunately, no further clinical examinations were performed due to the lack of compliance of the owner and to the death of the cat for causes unrelated to sarcoptic mange. This clinical case indicates that under certain circumstances, *S. scabiei* can reproduce and cause severe clinical signs in cats which are usually considered non-permissive hosts for this mite, and in which the disease is usually self-limiting. Clinic–pathological, epidemiological, and zoonotic implications are discussed.

## 1. Introduction

Cats can be infested by a range of mite species causing different clinical pictures and clinic–pathologic features, being *Otodectes cynotis*, *Notoedres cati*, and *Demodex* spp., the most important [1,2,3]. The worldwide-distributed burrowing mite *Sarcoptes scabiei* causes sarcoptic mange in several animal species and scabies in humans, but it has been very rarely described in cats [4,5]. This mite is characterized by a wide host adaptability, and its ancestor probably coevolved with humans, who have then transmitted it to domestic animals [5,6,7,8]. Accordingly, different variants have been described and taxonomically classified based on the host origin, e.g., *S. scabiei var. hominis*, and *S. scabiei var. canis* [5,9]. To date, a felid-associated variant has never been reported.

After mating on the host skin surface, female mites burrow tunnels in the superficial layers of the skin to lay 2–3 eggs per day. Mating takes place only once and females continue to lay eggs for the rest of their life (1–2 months). The larvae hatched from eggs molt into nymphs and then, to adults. When *S. scabiei* burrows in the host skin, an inflammatory and allergic response to their cuticle, eggs, feces, and saliva occurs, resulting in severe pruritic lesions [10]. The immune response and the histamine released in the injured tissues lead to a displacement of the mites and the expansion of the lesions [10]. If left untreated, sarcoptic mange causes generalized life-threatening lesions, severe general debilitation, and diffuse secondary bacterial infections [5].

The few known cases of sarcoptic mange in cats were characterized by mild non-pruritic crusted lesions to itching hair loss, scaling, crusting, and erythematous papules [4,7,11,12]. Importantly, no drugs are presently licensed in Europe for the treatment of this infestation in cats.

The present report describes a severe clinical case of sarcoptic mange in a cat and discusses epizootiological and clinic–pathological implications with the aim of improving knowledge on this parasitosis.

## 2. Case Description

A stray adult male domestic shorthair cat adopted from the street was referred to a veterinary practice in Rome (Italy) due to poor body conditions, diarrhea, and a severe dermatopathy, mainly localized on the head and the tail. The history of the cat was unknown.

The clinical examination revealed normal pulse and breath and cardiopulmonary auscultation, pale mucous membranes, a temperature of 38.2 °C, a body condition score of 2/5, and dull hair. The cat showed different skin lesions and cutaneous alterations (see Section 3).

The cat was hospitalized and subjected to different examinations, i.e., complete blood count, serum biochemistry, fecal flotation, rapid tests for feline immunodeficiency virus/feline leukemia virus and parvovirus (SNAP FIV/FeLV Combo test^®^ and SNAP Parvo test^®^ Idexx Laboratories), and a complete urinalysis. Moreover, multiple Scotch^®^ tests and skin scrapings performed both on the head and tail lesions were analyzed. Skin scrapings were performed by placing a drop of mineral oil on a sterile scalpel blade that was used to vigorously scrape the skin. The material obtained was placed on glass slides and covered with paraffin oil or a few drops of 10% potassium hydroxide solution. The preparations were observed under a microscope at 400X and 1000X magnifications. The Scotch^®^ test was performed by applying adhesive tape to the skin. The tape was then placed on a glass slide and observed under a microscope at 400X and 1000X magnifications.

Based on the results of the Scotch^®^ test and scraping examinations (see next section), the veterinarian treated the cat with a topical formulation containing esafoxolaner, eprinomectin, and praziquantel (Nexgard combo^®^ Boehringer Ingelheim), and with amoxicillin-clavulanic acid (20 mg/kg q12 per os) for a secondary bacterial infection. Probiotics were also administered for treating diarrhea. One month later, the skin condition of the cat markedly improved, and hair regrowth was observed. In the two months after, the cat was no longer clinically re-examined due to a lack of follow-up. The cat died due to a severe gastrointestinal disease unrelated to sarcoptic mange.

## 3. Results

### 3.1. Laboratory Results

Detailed laboratory alterations are listed in Table 1. The SNAP FIV/FeLV Combo test^®^ was positive for FIV, while the SNAP Parvo test^®^ was negative. *Dipylidium caninum* proglottids were detected in the macroscopic examination of the feces. Fecal flotation was negative.

### 3.2. Skin Lesions Description

**Figure 1:**
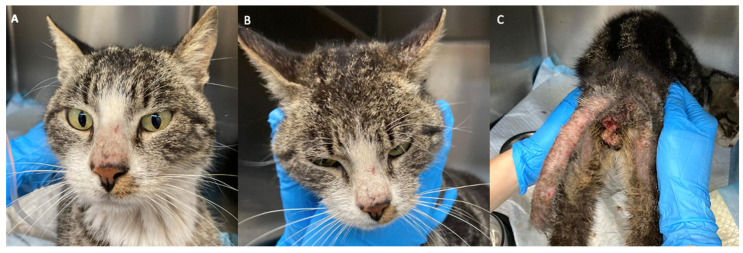
Skin lesions due to *Sarcoptes scabiei* on the nose (**A**), on the ears (**B**), and severe lesions on the caudal region (**C**).

The cat showed small excoriations on the nose and crusted lesions on the base of the ears, associated with slight alopecia (Figure 1A,B). The tail region had erythematous skin with severe alopecia. Scaling and exfoliation were present in these areas (Figure 1C). Partial-to-total bilateral alopecia was also observed in the posterior region of the thighs, with hyperpigmented skin, erythema, and hemorrhagic crusts at the base of the tail (Figure 1C).

During the hospitalization, no itching was observed, and neither was it reported by the owner. However, broken hair on the base of the tail (Figure 1C) observed in the trichoscopy suggested that the cat probably had pruritus and chewed or licked the hair.

### 3.3. Mites Description

**Figure 2:**
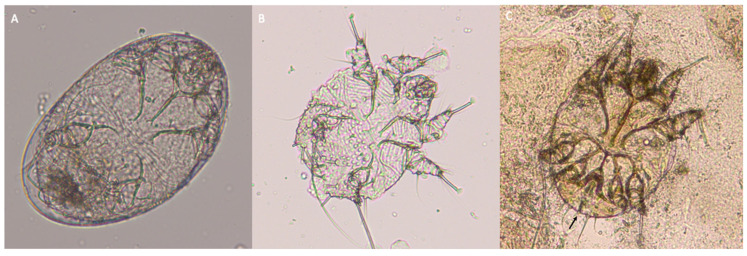
Egg (**A**), larva (**B**), and male adult (**C**) of *Sarcoptes scabiei* detected using skin scraping of the lesions. The terminal anus is indicated with the black arrow.

**Figure 3:**
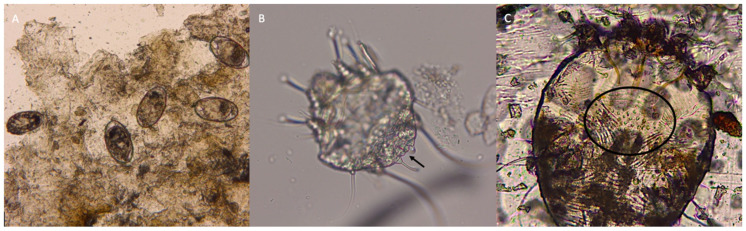
Several eggs of *Sarcoptes scabiei* retrieved in the skin scraping before the clarification (**A)**. The terminal anus (arrow) (**B**) and the dorsal cuticular spines are indicated (circle) (**C**).

Mite eggs, larvae, and adults were found in both the Scotch^®^ test and skin scraping (Figure 2 and Figure 3). The size of the adult mites ranged from 320–480 × 240–400 μm. The terminal anus and dorsal cuticular spines were clearly visible (Figure 2 and Figure 3). Mites were thus identified as *S. scabiei*. Mites morphologically and morphometrically compatible with *N. cati* were not detected.

## 4. Discussion

The present report describes a rare clinical case of sarcoptic mange in a domestic cat. In fact, despite data on *S. scabiei* in dogs being widely available, little information is known on clinical signs and diffusion of sarcoptic mange in feline hosts.

Although intense itching is a major clinical manifestation in dogs, in the present case pruritus was not observed during the hospitalization, despite broken hair in the caudal region suggesting that the cat had scratched using teeth. In the few published cases of sarcoptic mange in cats pruritus is occasional, unlike feline notoedric mange which causes severe itching [4,7,11,12,13].

The cat in the present study showed more severe and diffused skin lesions when compared with previously reported cases. In fact, in most of the few documented cases of feline sarcoptic mange, hair loss, scaling, and crusting localized in the head region were observed [7,12], with very few exceptions, e.g., mild erythematous lesions on the flanks and lateral thorax [4], mild crusty lesion on the tail [11], thick, wrinkled, scaly skin with extensive hair loss in the abdomen, proximal limb, and tail [14]. The severity of the present case could likely be attributed to a reduction in the efficiency of the immune response of the cat. In fact, its immune system was probably impaired due to FIV infection [7]. Interestingly, the heaviest form of human scabies, i.e., a crusted disease called “Norwegian scabies”, occurs in patients with impaired cell-mediated immunity due to HIV infection/AIDS or to immunosuppressive therapy [5,7,9,15,16].

When *S. scabiei* infects a non-permissive host, as in the case of *S. scabiei var. canis* infection in people, mites are not able to reproduce and the disease is usually mild and self-limiting. Despite the cat is considered a non-permissive host for *S. scabiei*, several eggs were detected in the skin scrapings (Figure 3). The evidence of *S. scabiei* mating was previously shown in another cat with severe crusted scabies and FIV infection [7], as here described. Thus, this report confirms that *S. scabiei* can also reproduce and cause a severe clinical presentation in non-permissive hosts when concomitant conditions impair their immune system.

The laboratory abnormalities here detected were nonspecific and not exclusively due to *S. scabiei*, as the cat was also infected with FIV. In previous cases, some laboratory alterations were described (i.e., leukocytosis with neutrophilia, and eosinophilia), but the majority of them were not comparable to those found in the present study (e.g., normal count of white cells with neutrophilia, lymphopenia, and monocytosis, possibly related to stress or inflammatory leukogram) [12].

In Europe, there are no licensed products for the treatment of sarcoptic mange in cats. The spot-on product used in this particular case, though not licensed for the treatment of feline sarcoptic mange, i.e., a formulation containing esafoxolaner/eprinomectin/praziquantel (Nexgard combo^®^ Boehringer Ingelheim, Ingelheim, Germany), is a recently commercially available endectocide labeled for the treatment of other feline ectoparasites, including *N. cati*. Broad-spectrum endectoparasiticides are advantageous for both owners and veterinary practitioners as, with a single product, animals with co-infestations can be successfully treated, as in the present case. Further studies are thus warranted to evaluate the efficacy of the here-used formulation against sarcoptic mange in cats, especially if this parasitosis will result more distributed than usually thought. Indeed, sarcoptic mange could be underestimated in clinical practice due to scarce awareness and hindrances inherent in the diagnosis, i.e., nonspecific clinical signs, possibly false negative results, or lack of morphological and morphometrical identification of mites retrieved in skin lesions. Thus, pets should be subjected to periodic and routine clinical checks, as the correct identification of mite species, along with the administration of proper treatment, allows cat and human health to be protected, considering the zoonotic potential of *S. scabiei* [17].

In conclusion, the present clinical case highlights the importance of considering *S. scabiei* in the differential diagnosis of feline skin diseases, especially in cats with an impaired immune system.

## Figures and Tables

**Table 1:** Alterations detected in the complete blood count, serum biochemistry, and urinalysis.

Complete Blood Count	Result	Reference Ranges
Segmented/mature neutrophils (*1000/μL)	15.97	2.3–12.6
Lymphocytes (*1000/μL)	0.6	0.7–7.9
Monocytes (*1000/μL)	1.21	0.07–1.2
**Serum biochemistry**	**Result**	**Reference Ranges**
AST [U/L]	54.97	5–40
Albumin [g/dL]	2.37	2.5–4.0
Urea [mg/dL]	84.36	20–65
Phosphorus [mg/dL]	5.26	1.6–5.0
**Urinalysis**	**Result**	**Reference Ranges**
pH	5.0	5.5–8.5
Protein to creatinine ratio	0.3	0.0–0.2

## Data Availability

Not applicable.
